# The Role of Glycosaminoglycans in Protection from Neonatal Necrotizing Enterocolitis: A Narrative Review

**DOI:** 10.3390/nu12020546

**Published:** 2020-02-20

**Authors:** Kathryn Burge, Erynn Bergner, Aarthi Gunasekaran, Jeffrey Eckert, Hala Chaaban

**Affiliations:** Department of Pediatrics, Division of Neonatology, University of Oklahoma Health Sciences Center, 1200 North Everett Dr., ETNP7504, Oklahoma City, OK 73104, USA; Kathryn-Burge@ouhsc.edu (K.B.); Erynn-Bergner@ouhsc.edu (E.B.); Aarthi-Gunasekaran@ouhsc.edu (A.G.); Jeffrey-Eckert@ouhsc.edu (J.E.)

**Keywords:** necrotizing enterocolitis, inflammation, neonatal, intestine, prematurity, human milk, glycosaminoglycans

## Abstract

Necrotizing enterocolitis, a potentially fatal intestinal inflammatory disorder affecting primarily premature infants, is a significant cause of morbidity and mortality in neonates. While the etiology of the disease is, as yet, unknown, a number of risk factors for the development of necrotizing enterocolitis have been identified. One such risk factor, formula feeding, has been shown to contribute to both increased incidence and severity of the disease. The protective influences afforded by breastfeeding are likely attributable to the unique composition of human milk, an extremely potent, biologically active fluid. This review brings together knowledge on the pathogenesis of necrotizing enterocolitis and current thinking on the instrumental role of one of the more prominent classes of bioactive components in human breast milk, glycosaminoglycans.

## 1. Introduction

Necrotizing enterocolitis (NEC) is a common intestinal inflammatory disorder developing during the neonatal period. The disease progresses rapidly from subtle abdominal distension to necrosis, intestinal perforation, multi-organ failure, and, in severe cases, death [1,2]. Because of better survivorship among the smallest premature infants [3], as well as a dearth of treatments for the disease [4], the incidence and health burden of NEC have only grown in recent decades [1]. Mortality rates often approach 30% in infants born less than 1500 g, and range higher in those babies requiring surgical intervention [1]. Infants surviving NEC often suffer from long-term morbidities related to both the disease and its treatment, including neurodevelopmental delays, retinopathy, and short-bowel syndrome [5,6].

Although the exact etiology of the disease is still unclear [7], a number of risk factors have been identified. These include, among others, prematurity and low birthweight status, developmental immaturity of both the intestine and immune system, inappropriate microbial colonization of the gut, and formula feeding [8]. What few advances have been made in NEC treatment recently revolve around the growing understanding of the importance of optimal infant nutrition, particularly that provided by human breast milk (HM), sourced either from the mother or a donor [5,9,10].

HM functions in several critical biological roles in neonates, including support for intestinal development and maturation, protection against pathogens, and basic dietary sustenance for growth [9,11]. In babies fed exclusively HM, enteric infections are reduced by approximately 50% compared to infants fed bovine-based formula [12,13]. In the preterm population specifically, infants fed exclusively HM, as opposed to at least partial bovine-based feedings, experience significant reductions in morbidity and mortality [14]. In very low birthweight (VLBW, <1500 g) infants, feedings composed of at least 50% HM in the first two weeks of life correlate with a six-fold decrease in the incidence of NEC [15]. Additional studies [16,17,18] have also indicated HM, particularly that sourced from the biological mother, reduces the incidence and severity of NEC in preterm infants. Donor milk, while arguably preferable to bovine-based formula [19], loses effectiveness through the pasteurization process, and is also not age-matched to the developmental stage of the infant to whom it is donated [20,21]. Thus, studies utilizing donor HM have shown mixed results when examining utility in protection against preterm pathogens, NEC, and mortality [22,23,24].

This narrative [25,26] review briefly summarizes what is known about the pathogenesis of NEC in premature infants, and expands upon the potential role of glycosaminoglycans, bioactive components of HM, in protection from NEC.

## 2. Pathogenesis of Necrotizing Enterocolitis

The pathogenesis of NEC appears to be highly complex and multifactorial. Data predominantly implicate developmental immaturity of the intestinal immune system [27], as well as an altered microbiome [28,29], in the development of a dysfunctional intestinal barrier [30] in necrotizing enterocolitis. While the sequence of events in NEC etiology remains unclear, the disease is likely initiated by an excessive stimulation of toll-like receptor 4 (TLR4) by Gram-negative bacteria [31] in the ileum of the premature infant. Activation of this receptor [32,33,34,35,36,37,38] leads to extensive inflammation, denoted by apoptosis of enterocytes along the luminal border, impaired replacement of these enterocytes, increased release of proinflammatory cytokines and chemokines, and, in total, breakdown of the intestinal barrier [39,40,41]. This impaired intestinal barrier allows for greater bacterial translocation [42], leading to increased inflammation via direct contact of pathogenic bacterial antigens with the mucosal immune system [43]. Neutrophil recruitment to the intestinal border and production of reactive oxygen species (ROS) further contributes to this inflammation [1,44,45]. TLR4 activation of the underlying endothelium initiates microvascular complications, including a reduction in endothelial nitric oxide synthase (eNOS), resulting in intestinal ischemia and necrosis [38,46,47]. Altogether, a positive feedback loop of inflammation is created, overwhelming any counterregulatory attempts by the host. Inflammation spreads systemically, leading to full-blown NEC and complications in organs as distant as the brain [48]. Our understanding of the clinical picture in NEC is muddied by a lack of appropriate animal models through which researchers can replicate most aspects of the human condition, and subsequent inability to translate findings in these animal models to the bedside. In particular, our limited understanding of the immature innate and adaptive immune systems and developing microbiome, and potential interplay of these two factors, hinders abilities to target effective treatments for NEC.

### 2.1. Developmental Immaturity

#### 2.1.1. Innate Immune System

The innate immune system in the small intestine is comprised of both a physical barrier of intestinal epithelial cells (IECs) and their biochemical products, and an underlying and complementary immunological barrier [49]. The physical barrier is often considered to include intestinal alkaline phosphatase, a loose layer of mucus [50], tight junctions linking IECs, and antimicrobial proteins (AMPs) released by a specialized lineage of IECs, Paneth cells [51].

A number of developmental differences in IECs and the innate intestinal immune system have been associated with the pathogenesis of NEC. For example, goblet cell numbers and levels of their signature mucin, Muc2, are reduced in both mouse models and premature human infants with NEC [32], likely associated with developmental immaturity of the ileum [52]. Reductions in goblet cell numbers are thought to contribute to increased severity and incidence of NEC [52,53], potentially due to increased levels of bacterial translocation across an epithelium now inadequately guarded by mucus [53,54]. In addition, Paneth cells in premature infants are deficient both in number and function [55], altering the levels of lysozyme and defensins [56,57] and likely contributing to the development of NEC via associated changes in the microbiome [58].

Immune cells in the intestines of premature infants often appear to function differently than those of term neonates and adults, predisposing these infants to the development of NEC. Neutrophils, first responders to tissue injury, demonstrate impaired phagocytic ability, increased oxidative burst products, and variable cytokine production in premature infants compared to term babies [59]. Intestinal macrophages in preterm infants appear to be hyperreactive and produce excessive proinflammatory cytokines [60,61,62]. In addition, dendritic cell morphology and functionality in preterm neonates appears to differ from that of term babies [63], and in a mouse model of experimental NEC, the recruitment of dendritic cells to the luminal border directly disrupts intestinal barrier function [64].

Alterations in inflammatory signaling also predispose premature infants to the development of NEC by creating a host environment hyperreactive to both commensal and pathogenic organisms [33,65]. TLR4, specifically, is thought to play a crucial role in NEC [66] due to its abnormally increased expression in prematurity, both in mice and human infants [67,68]. Increased TLR4 expression appears to precede histological damage of the intestine in mouse NEC models, strongly implicating a role for TLR4 in the pathogenesis of the disease [65,69].

#### 2.1.2. Adaptive Immune System

Adaptive immunity in the small intestine, thought to be less effective than innate immunity in a newborn [70], is dependent upon antigen-presenting cells (APC), primarily dendritic cells and IECs. A number of differences in adaptive immune function exist in infants with NEC, many of which are likely a developmental artifact of prematurity. Levels of secretory immunoglobulin A (sIgA), an antibody produced by lamina propia B cells and recognized for its ability to maintain the microbiome by neutralizing pathogenic bacteria [71], are reduced in premature infants compared to term babies [72,73], with clear implications for the development of NEC. Additionally, a significant role for T cells in the pathogenesis of NEC is becoming increasingly evident. For example, mice lacking functional T and B cells are less susceptible to NEC, but transfer of functional T cells to these animals increases susceptibility to the disease [34].

Intraepithelial lymphocytes (IELs), regulators and initiators of both innate and adaptive immune responses to bacterial invasion [74], are dispersed within the intestinal epithelium [41]. The γδ subset of IELs, created during embryogenesis, are more reactive in the neonate than in the adult [75], and preterm infants with NEC show significantly lower levels of these specialized T cells compared to healthy preterm babies [76]. Regulatory T cells (Tregs), T cells modulating the immune response and promoting tolerogenicity, are decreased in both experimental mouse [34] and human [77] NEC. In neonates, baseline levels of the Treg inhibitor STAT3 (signal transducer and activator of transcription 3) are increased compared to those of adults [34]. In a mouse model of NEC, when a STAT3 inhibitor is introduced, levels of Tregs increase and NEC severity is reduced [34].

Finally, T helper (Th) cell differentiation also appears to be dysregulated during NEC. In particular, Th17 cells, characterized by production of the inflammatory interleukin 17A (IL-17A) cytokine, have been shown to be upregulated in both murine and human NEC, and are thought to play a role in intestinal barrier dysfunction [34]. In an experimental model of NEC, mice treated with all-trans retinoic acid (ATRA), an inhibitor of Th17 differentiation [78], showed lower levels of Th17 cells, increased populations of Tregs, and reduced NEC severity [34,79]. Interestingly, retinoic acid is produced endogenously by IECs, but this production is thought to be largely dictated by luminal commensal bacteria [80].

### 2.2. Dysbiosis

Colonization of the infant intestine, previously thought to commence at birth, may originate in the placenta [81], where the fetus is possibly surrounded by non-sterile amniotic fluid [82], though this finding has been recently debated [83,84]. The main event responsible for infant intestinal colonization, however, is likely birth [85]. Vaginal delivery of term infants results in initial colonization with predominantly aerobic bacteria, including *Streptococcus*, *Staphylococcus*, and *Lactobacillus* [86]. As these aerobic bacteria consume oxygen, the microbiome shifts to reflect greater populations of facultative anaerobes, followed by strict anaerobes such as Clostridia and Bifidobacteria species [87,88]. These obligate anaerobes produce short-chain fatty acids (SCFAs), anti-inflammatory lipids known to regulate epithelial and immune cell development in the gut [89], and protect against the proliferation of pathogenic bacteria [90]. In preterm infants, the development of the intestinal microbiome following birth appears to follow a reasonably predictable progression from Bacilli to Gammaproteobacteria to Clostridia [91]. The resulting intestinal population in preterm infants is characterized by lower diversity, fewer species numbers, and a greater proportion of pathogenic bacteria, many of which could initiate the TLR4 signaling cascade via lipopolysaccharide (LPS), compared to that of infants born at term [92,93,94,95]. This errant microbiome in the premature infant, together with an immature intestinal immune system, presents a mechanism for hyperinflammation and deterioration of the critical intestinal barrier.

Dysbiosis can refer to improper proportions of microbial species, as well as a lack of diversity and richness of species overall [96]. A skewed microbiome can also result from the gain or loss of critical microbial populations, often negatively affecting the functionality of both the intestine and its interwoven immune system. An appropriate microbiome is thought to be indispensable in triggering the maturation of the mucosal immune system in the gut [97]. Support for the role of dysbiosis in NEC is largely derived from studies in germ-free animals, in which the disease cannot be reproduced [98,99,100]. Additionally, factors indirectly influencing microbial colonization in the infant, such as antibiotic use in the mother [67], can increase NEC development risk in the infant. While a single pathogen is not thought to induce NEC, a series of microbial shifts in the microbiome has been associated with development of the disease [28], and these changes usually precede diagnosis [101], implicating a potential role for dysbiosis in the pathogenesis of NEC. For example, infants with NEC often have reduced populations of Bifidobacteria, Bacteroidetes, and Firmicutes anaerobes, particularly Negativicutes, and increased levels of Proteobacteria and Actinobacteria [28,101,102,103,104,105]. This reduction in anaerobes in NEC leads to a decline in the production of protective SCFAs [7,103,104], a further complication of NEC-associated dysbiosis. Generally, the microbiome of infants developing NEC appears to be characterized by reductions in both species richness and diversity [95,106,107], though not all studies have noted these trends [101,105,108].

A number of factors beyond prematurity can influence the microbial colonization of the infant intestine. The use of antibiotics, rampant in the premature infant population [109,110], is known to increase the risk of NEC development, with risk correlating strongly to duration of treatment [111,112]. Antibiotic exposure in neonates may lead to increases in Proteobacteria, decreases in Actinobacteria, and, as with all antibiotic usage, inadvertent selection for antibiotic-resistant strains [85,113,114,115]. Mode of delivery also strongly influences the development of the infant microbiome. Babies born via caesarean section are often colonized by increased populations of *Clostridium* and *Staphylococcus* and decreased levels of *Bifidobacterium* and *Bacteroides* compared to infants born vaginally [102,116,117]. Antacid use, particularly histamine-2 (H_2_) blockers, can disrupt the acid-base balance in the premature intestine [118], predisposing the infant to NEC [119,120] by favoring populations of Proteobacteria over those of Firmicutes [121,122]. Even endogenous factors may affect the relative proportions of intestinal colonizers. For example, Paneth cell lysozyme and defensin secretion patterns, altered in premature infants [56,57], can lead to irregular microbial colonization in infants [58,123]. Finally, mode of feeding can direct the development of the neonatal microbiome. HM contains a microbiome of its own [124], likely specialized for the infant with whom it is associated [125,126], and thus may be uniquely protective. While breastfeeding stimulates the expansion of *Bifidobacterium*, in particular, and inhibits the growth of pathogenic bacteria [127,128], formula feeding often leads to a slightly more diverse assemblage of Enterobacteriaceae, *Bacteroides*, *Lactobacillus*, *Prevotella*, and, especially, *Clostridium* [87,129,130,131]. A number of biological components of HM are thought to help shape the development of the infant microbiome, as well as prime intestinal immune development and maturation.

## 3. Glycosaminoglycans in Milk

HM is a complex mixture of biologically active molecules known to play a role in infant nutrition, protection from pathogens, and development and maturation of the intestinal immune system. The composition of HM is not static, changing over time to meet the needs of a growing infant. Colostrum, the first milk, is high in minerals, vitamins, hormones, and growth factors [132]. Transitional milk replaces colostrum at approximately one week postpartum, and is high in fat and lactose [133]. Finally, mature milk follows at two weeks postpartum, consisting largely of water and nutritional macronutrients necessary for infant growth [134]. All stages of HM, however, contain various compounds necessary for development of the microbiome and protection of the infant from pathogens. For example, oligosaccharides, commonly referred to as human milk oligosaccharides (HMOS), are found in large quantities in HM and are largely unabsorbed, serving as prebiotics for commensal gut bacteria [5], thereby bolstering and developing the innate immune system and microbiome in neonates [135,136]. HM also contains immunoglobulins, such as sIga, which potentially serve as prebiotics capable of assisting in the proper colonization of the newborn gut [137] while inhibiting growth of pathogenic bacteria [138]. Glycosaminoglycans (GAGs), a class of polysaccharides found in the extracellular matrix and outer surface of cells, are also prevalent in HM. Our understanding of the potential functions of this class of molecules is still evolving, but their elevated concentrations in early HM and a number of studies indicating protective capabilities of these molecules against pathogens may indicate importance in the prevention of NEC.

**Figure 1:**
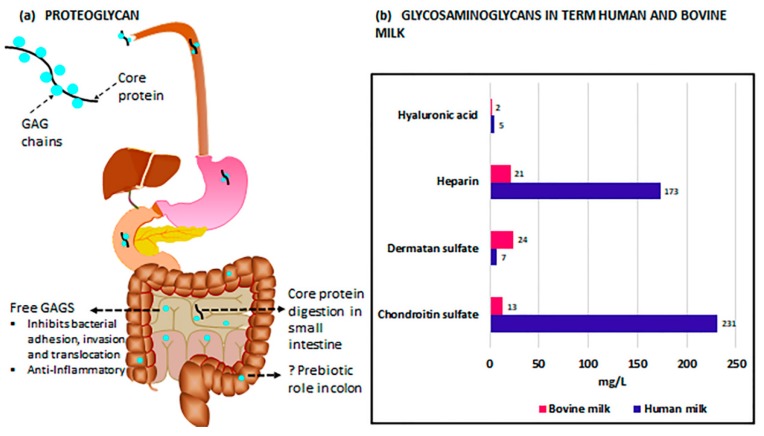
Glycosaminoglycans, found in much greater quantities in human breast milk (HM), traverse the intestines largely untouched: (**a**) Digestion and potential function of glycosaminoglycan-associated proteoglycans in milk. (**b**) Comparison of total glycosaminoglycan content of term human and bovine milk. GAG: glycosaminoglycan; GAGS: glycosaminoglycans.

GAGs, molecules composed of repeating, often highly sulfated disaccharides, include heparin, heparan sulfate (HS), keratan sulfate (KS), hyaluronic acid (HA), chondroitin sulfate (CS), and dermatan sulfate (DS) [139]. In HM, these GAGs, with the exception of HA, are bound to a protein core and expressed as proteoglycans [140]. HA, uniquely, is neither sulfated nor assembled bound to a protein core [141]. Once in the small intestine, pancreatic enzymes digest the proteins, resulting in free GAGs (Figure 1a). Due to a lack of endogenous host enzymes in the small intestine capable of further breaking down free GAGs [142], these molecules remain largely undigested through the majority of the gastrointestinal tract [143], with eventual breakdown likely occurring in the cecum or colon [142]. In the case of HA, and potentially other GAGs, the resulting fragment sizes, whether created by endogenous breakdown of the parent molecule or intentional supplementation of a specific molecular weight, may differ vastly in function [144,145].

The composition of GAGs in milk differs greatly depending upon the source. In term HM, GAGs, in sum, are approximately seven times more prevalent than in bovine milk, the basis of most infant formulas [146]. Coppa et al. [146] determined, via a variety of methods, that large differences in GAG relative composition also exist between the two milks (Figure 1b). CS accounts for 55% of the term HM GAGs compared to only 21% of GAGs in bovine milk, but because of the reduced total quantity of GAGs in bovine milk, this amounts to nearly 23 times as much CS in term human compared to bovine milk. Term HM also has substantially higher levels of heparin (173 mg/L compared to 21 mg/L) and HA (5 mg/L compared to 2 mg/L) and lower levels of DS (7 mg/L compared to 24 mg/L) than bovine milk, though, interestingly, bovine milk is higher in HA by percentage (4.5% compared to 1.3%). Additionally, GAGs present in term HM appear to be generally less sulfated compared with those in bovine milk [146], though any impact of this difference on the infant has not been explored.

A disparity in bioavailability also appears to exist between human and bovine milk. Maccari et al. [143] compared the residual GAG content of the feces of term infants fed either HM or formula, and noted significantly lower recovery of HM GAGs compared to those from formula, indicating a much greater utilization of the GAGs derived from HM. In addition, a greater proportion of highly sulfated GAGs, present at higher levels in bovine milk compared to HM, appeared in the feces of both groups, potentially suggesting an inability of distal intestinal bacteria to catabolize these compounds [147]. Differences in milk glycosaminoglycan composition, overall quantity, and bioavailability may underly some of the protective effects of breastfeeding with regard to NEC pathogenesis.

Notably, gestational age of the infant at birth and stage of lactation have prominent influences on the GAG composition of HM. Coppa et al. [148] demonstrated that while preterm and term HM have consistent proportions of the two foremost GAGs, chondroitin sulfate and heparin, the total GAG content is approximately three times higher in preterm milk. The respective percentages of CS and heparin are maintained as total GAG levels vary over the first month of lactation, with peak levels of GAGs on day 4 of colostrum (9.3 g/L and 3.8 g/L in preterm and term HM, respectively) and a subsequent decline to the end of the month (4.3 g/L and 0.4 g/L in preterm and term HM, respectively). Interestingly, 50% (preterm) and 73% (term) of this reduction in GAG content is noted to occur between days 4 and 10 [148]. Wang et al. [149] have established this progressive decrease of HM GAG content occurs through at least the first six months of lactation. Additionally, differences in the degree of sulfation occur during the lactation period, with HS sulfation increasing slightly over the first six months and CS sulfation peaking at day 43 before subsequently declining [149]. While the physiological rationale underlying GAG sulfation variability during the breastfeeding period has not yet been appreciated, these changes in sulfation patterns are likely functionally significant [150].

Maternal characteristics may also alter both the GAG composition of HM and the ability of the infant, indirectly, to break down those GAGs in the distal intestine. While Volpi et al. [151] did not find GAG compositional changes among milk samples from mothers of varying ethnicities, Mannello et al. [152] noted maternal health could directly influence GAG composition, as alterations in the structure and sulfation levels of CS in the milk of a breast affected by invasive carcinoma differed with those in the unaffected breast of the same mother. Finally, Cerdό et al. [153] have established that the microbiome of infants born to obese mothers is more capable of glycosaminoglycan degradation compared with that of infants born to mothers of normal weight, with unspecified effects on the risk of developing NEC. Further investigation into the potential impact of maternal obesity on GAG content of HM and the ability of infants born to obese mothers to utilize HM GAGs would be of interest.

The utility of donor milk as a suitable substitute for formula has often been questioned, particularly given the pasteurization process is known to reduce the bioactivity and concentration of critical immunoglobulins, growth factors, and digestive enzymes [19]. HM contains active glycosidases, enzymes capable of degrading glycoconjugates such as glycosaminoglycans in a time- and temperature-dependent manner; however, these enzymes are unlikely to significantly alter the composition of GAGs in donor milk, as little breakdown in glycoconjugates is seen with storage at 37 °C for up to 16 h [154]. Additionally, Coscia et al. [155] subjected donor milk samples to Holder pasteurization, a method often utilized by milk banks, and noted the concentration and proportions of HM GAGs are not significantly altered by the process. Thus, the current preference for donor milk over formula may be warranted in this context, especially in at-risk, preterm infants, as GAG concentrations and relative proportions remain largely unaffected by common storage and processing techniques.

## 4. Glycosaminoglycan Protection against NEC

**Figure 2:**
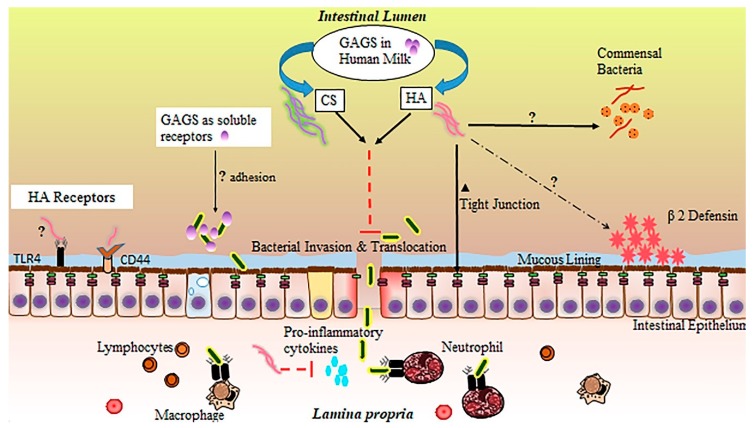
Schematic of potential glycosaminoglycan mechanisms of protection in necrotizing enterocolitis (NEC). CS: chondroitin sulfate; HA: hyaluronic acid; GAGS: glycosaminoglycans.

In the small intestine, GAGs are believed to participate in a number of biological processes, including molecular trafficking, maturation, and differentiation of a variety of cell types, modulation of inflammatory events, structural support, and adhesion to bacteria in the intestinal lumen [156,157,158,159]. Importantly, GAG incorporation into the extracellular matrix and epithelial cell surface is thought to be essential to a functional intestinal barrier [160]. Inflammation, particularly driven by proinflammatory cytokine release, has been shown to disrupt endogenous production of sulfated GAGs [161]. In NEC, impaired distribution of intestinal GAGs appears to mirror the patchy, skip lesion nature of the disease [162]. While GAGs are not digested and incorporated into small intestinal tissue [143], their supplementation through HM or other means may still provide significant benefits to the neonatal small intestinal epithelium through their interaction with the epithelial surface or luminal contents [163], especially in the context of their potential loss during inflammation. These extracellular interactions of GAGs with IECs or luminal bacteria likely contribute substantially to the protective effects of HM against NEC. While the precise function of GAGs in HM is incompletely understood (Figure 2), studies attributing protective effects to individual GAGs, sourced either from HM extractions or biosynthetic preparations, are accumulating, with the large majority focusing on CS, HA, and heparin.

Generally, GAGs are believed to exhibit antiviral and antibacterial properties [164]. HA has been demonstrated to inhibit bacterial growth [165,166], and is a common matrix component of bioengineered orthopedic scaffolding because of this bacteriostatic property. HM GAGs may also influence the composition of the neonatal microbiome beyond growth constraints applied to certain microbial species, resulting in a wide variety of potential physiological effects. Recently, several species of human commensal bacteria, including strains associated with common probiotic formulas, have been shown to actively degrade host GAGs in the intestine [167], lending some credence to the idea that HM or formula-supplemented GAGs could act as prebiotics [168], promoting the growth of only those commensal species outfitted with the enzymes necessary to metabolize these compounds. While these prebiotic effects would be far more likely to affect the distal intestine as opposed to the ileum, the ramifications of this potential GAG influence on the microbiome in total may include changes in NEC susceptibility and require further investigation.

GAGs are also known to reduce microbial adhesion to IECs, often the initial step in infection. Sava et al. [169] pretreated Caco-2 colonocytes with a mixture of heparin, HS, and CS, reducing the capacity of Enterococci bacteria to adhere to the host cell surface. Hafez et al. [170] demonstrated similar findings with regard to Staphylococci adhesion to host epithelial cells in the presence of free GAGs. Treatment of HT-29 colorectal cells with heparin, a prominent GAG in HM, has also been shown to reduce internalization of a number of bacterial species via a reduction in cellular adhesion [171]. Antimicrobial characteristics of GAGs have also been shown to extend to those isolated directly from HM. For example, Newburg et al. [172] demonstrated CS or a CS-like compound extracted from HM can inhibit binding of the gp120 human immunodeficiency virus (HIV) envelope protein to its receptor, an essential early step in transmission of the virus. Coppa et al. [140] treated intestinal cell lines with GAGs extracted from HM and demonstrated a reduction in pathogenic bacterial binding of intestinal receptors, while Hill et al. [173] demonstrated the ability of HA 35 (biosynthetic HA of an intermediate 35 kDa size and with qualitatively similar effects to that of the inclusive HM HA fraction) to limit intestinal adhesion of *Salmonella* Typhimurium.

GAGs present in HM may also work synergistically with the host immune system to both upregulate endogenous defenses and tame the type of destructive, runaway inflammation characteristic of NEC [174]. HA fragments of varied sizes have been shown to have protective effects on the intestinal epithelium. Zheng et al. [175] noted exogenous administration of 750 kDa HA fragments ameliorated disease in a colitis mouse model through a TLR4- and cyclooxygenase-2 (COX-2)-dependent repair of the epithelium, while Riehl et al. [176] found similar protective effects in irradiated small intestinal tissue. HA of 900 kDa size has also been shown to protect the intestinal epithelium of immunocompromised mice through a reduction in inflammatory signaling [177]. Additionally, both HA 35 and a polydisperse HM HA extract upregulate the antimicrobial protein human β-defensin 2 (HβD2) in human intestinal epithelial cells and its ortholog in the murine large intestine [173,178].

Our group recently demonstrated the effectiveness of HA 35 in reducing the incidence and severity of disease in a mouse model of necrotizing enterocolitis. Gunasekaran et al. [179] treated mouse pups (age P14–16) with HA 35 (15 mg/kg or 30 mg/kg) once per day for three days prior to the initiation of NEC. NEC was induced using a two-hit model of intraperitoneal dithizone injection followed by oral administration of *Klebsiella pneumoniae* [55]. A stark reduction in proinflammatory cytokine release (tumor necrosis factor-alpha (TNF-α), GRO-α (growth-regulated oncogene-alpha), IL-12p70, and IL-6) was seen with HA 35 treatment (either dose) compared to untreated NEC. These changes, coupled with upregulation in tight junction proteins, likely led to the reduction in pathological intestinal permeability and associated bacteremia, ultimately resulting in significantly improved pathology of the ileum, substantially diminished disease severity, and significantly greater pup survival.

CS also exerts a number of well-documented effects on inflammation, including reductions in pro-inflammatory cytokine and NF-κB (nuclear factor kappa-light-chain-enhancer of activated B cells) levels [180], weakened COX-2 and NOS-2 (nitric oxide synthase-2) activities [181], and the upregulation of a variety of antioxidant enzymes [182]. CS has demonstrated positive impacts in intestinal bowel disease (IBD) [183,184], likely through anti-inflammatory effects [185] and increased epithelial and mucosal tissue repair [183]. The anti-inflammatory effects of heparin, including reductions in pro-inflammatory TNF-α and IL-6 signaling [186], have also been demonstrated in the context of intestinal inflammatory diseases. Often utilized as a first line of treatment in IBD [187], heparin, specifically low-molecular-weight or unfractionated heparin, has been shown to ameliorate disease activity through a combination of anti-inflammatory and anticoagulative effects [188], resulting in increased mucosal healing and improved intestinal barrier function [189].

Altogether, glycosaminoglycan-associated reduction in pathogen binding to host IECs, an upregulation in intestinal defenses by GAGs, and a reduction in excessive inflammatory signaling is likely to lead to an improvement in intestinal barrier function and a significant decline in bacterial invasion and translocation, critical events in the pathogenesis of NEC [190,191]. Hall et al. [192] established a line of goblet-like cells are more susceptible to invasion when bacteria are freely suspended in bovine-based formula compared to HM. Hill et al. [173] demonstrated a polydisperse HM HA extract is capable of protecting colonocytes from *Salmonella* infection in vitro. Mice treated with HA 35 are protected from *Citrobacter rodentium* infection via an upregulation in the critical tight junction protein, ZO-1 (zonula occludens-1), resulting in reduced intestinal permeability and inhibited bacterial translocation across colonic epithelium [193], similar to our findings in a murine NEC model [179].

**Figure 3:**
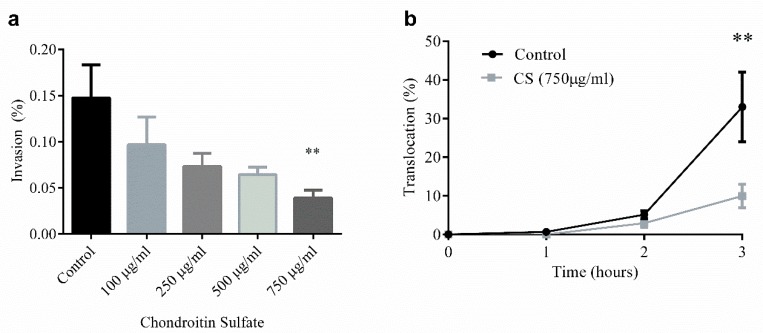
The effects of chondroitin sulfate on *E. coli* invasion and translocation of T84 colonocyte monolayers: (**a**) chondroitin sulfate (CS) was given prophylactically for 48 h prior to infection. A dose-dependent reduction in bacterial invasion occurred with CS (M ± SEM), with 750 µg/mL showing significantly lower bacterial invasion compared to control (** *p* = 0.0071; eta-squared = 0.0944); (**b**) CS at 750 µg/mL was significantly protective by the third hour of inoculation (M ± SEM, ** *p* = 0.0018). (Reprinted with permission of authors and SAGE Publishing [194]).

Our group has also directly interrogated the ability of CS, the most common GAG in HM [146], to limit both bacterial invasion (Figure 3a) and translocation (Figure 3b) in T84 colonocyte monolayers, an in vitro model of the intestinal epithelium [194]. CS, at a concentration of 750 µg/mL given prophylactically for 48 h prior to bacterial challenge, reduces invasion and translocation of SCB34 *Escherichia coli*, an invasive, multi-drug resistant bacterial strain isolated from a neonatal early-onset sepsis case [195], by 75% compared to control. In this study, CS shows no effects on cell viability while also reducing, though not significantly, the production of the inflammatory chemokine, IL-8. Given the potent effects of GAGs on inflammation and prevention of infection, the availability of these compounds in HM, and potentially their supplementation in formula following further systematic review, may be critical to neonatal health in general, and specifically, in the prevention of NEC.

## 5. Conclusions

In this review, we assessed the GAG composition of sources of neonatal nutrition and relative changes across the duration of infant feeding, as well as summarized the potential protective effects of these GAGs against necrotizing enterocolitis. As common components of HM, GAGs are receiving increased attention because of their demonstrated antimicrobial and anti-inflammatory effects, and their potential to ameliorate intestinal inflammation and associated bacterial translocation of the epithelium. The protective effects on host barrier function, combined with beneficial interactions with, and positive influences on, luminal bacteria, likely serve to strengthen innate defenses against gastrointestinal infection in the neonate. Additional studies are needed to further characterize the effects of HM-derived GAGs on the intestinal epithelium, their interactions with specific bacteria, and their influence on the neonatal intestinal microbiome in full, particularly in the contexts of prematurity and NEC.
